# First basic performance evaluation of the XE-2100 haematology analyser

**DOI:** 10.1155/S1463924699000152

**Published:** 1999

**Authors:** Izumi Tsuda, Masayuki Hino, Takayuki Takubo, Tomoko Katagami, Hiroshi Kubota, Seiki Kawai, N. Tatsumi

**Affiliations:** 1 Department of Clinical and Laboratory Medicine Japan; 2 Department of Clinical Hematology Japan; 3 Central Clinical Laboratory Osaka City University Medical School Japan; 4 Wakakoukai Hospital Japan

## Abstract

The newly developed XE-2100 haematology analyser can provide complete blood counts, leukocyte differentials, perform reticulocyte analysis, and obtain quantitative data on nucleated red blood cells (NRBCs). In this study, we evaluated the basic performance of this instrument using routinely obtained blood specimens treated with ethylenediaminetetraacetic acid-2k. Reproducibility, carryover, stability during storage at 4°C and room temperature, and accuracy were evaluated. In this evaluation, reproducibility was good and little carryover was found. Accurate measurements were possible for up to 48h of storage. A good correlation between findings with the XE-2100 and SE-9000 haematology analysers was found for complete blood count on 210 samples tested. The leukocyte differential obtained with the XE-2100 correlated well with eye counts and with the results obtained with the SE-9000 automated haematology analyser, with r values over 0.9 for the percentages of neutrophils, lymphocytes and eosinophils. The precision and accuracy of VRBC and reticulocyte counts by the XE-2100 were satisfactory. We used the XE-2100 to obtain differential counts for bone marrow aspirates, and good correlations with manual differentials were obtained for total nucleated cell count, percentage of myeloid cells and percentage of erythroid cells. The performance of the XE-2100 was excellent, and this instrument should be able to provide reliable data to clinical laboratories.


Journal of Automated Methods & Management in Chemistry, Vol. 21, No. 4 (July-August 1999) pp. 127-133

### First basic performance evaluation of the XE-2100 haematology analyser

**Izumi Tsuda,[1] Masayuki Hino,[2] Takayuki Takubo,[1] Tomoko Katagami,[3] Hiroshi Kubota,[3] Seiki Kawai[4] and N. Tatsumi[1,2]**

[1] *Department of Clinical and Laboratory Medicine,* [2] *Department of Clinical Hematology,* [3] *Central Clinical Laboratory, Osaka City University Medical School, and* [4] *Wakakoukai Hospital, Japan. e-mail: noritatsumi@med.osaka-cu.ac.jp*

*The newly developed XE-2100 haematology analyser can provide complete blood counts, leukocyte differentials, perform reticulocyte analysis, and obtain quantitative data on nucleated red blood cells (NRBCs). In this study, we evaluated the basic performance of this instrument using routinely obtained blood specimens treated with ethylenediaminetetraacetic acid-2K. Reproducibility, carryover, stability during storage at 4°C and room temperature, and accuracy were evaluated. In this evaluation, reproducibility was good and little carryover was found. Accurate measurements were possible for up to 48h of storage. A good correlation between findings with the XE-2100 and SE-9000 haematology analysers was found for complete blood count on 210 samples tested. The leukocyte differential obtained with the XE-2100 correlated well with eye counts and with the results obtained with the SE-9000 automated haematology analyser, with r values over 0.9 for the percentages of neutrophils, lymphocytes and eosinophils. The precision and accuracy of NRBC and reticulocyte counts by the XE-2100 were satisfactory. We used the XE-2100 to obtain differential counts for bone marrow aspirates, and good correlations with manual differentials were obtained for total nucleated cell count, percentage of myeloid cells and percentage of erythroid cells. The performance of the XE-2100 was excellent, and this instrument should be able to provide reliable data to clinical laboratories.*

### Introduction

New haematology analysers have recently been developed, and each has been claimed to improve laboratory efficiency by increasing automation capabilities or by the addition of new parameters that could not previously be automatically determined. The XE-2100 haematology analyser (Sysmex, Kobe, Japan) was developed to meet the needs of present and future clinical laboratories. It can provide maximal information to haematology laboratories, including simultaneous complete blood counts, leukocyte differential counts, and reticulocyte analytes. It is capable of determining a new parameter, the count of nucleated red blood cells (NRBCs), not obtainable with previous Sysmex instruments. Its high throughput will have a beneficial impact especially on large-sized laboratories required to analyse increasing numbers of specimens. In this study, we performed a basic evaluation of the XE-2100.

### Materials and methods

### *Materials*

Venous blood treated with ethylenediaminetetraacetic acid-2K was processed within 6 h after blood collection, except for stability tests. The samples were selected from the laboratory routine workload except when otherwise indicated.

### *Determination of parameters by the XE-2100*

The principle of determination of the complete blood count is the same as that used by the Sysmex SF-3000, a blood cell counter with semi-conductor laser light [1] performing haemoglobinometry based on the sodium lauryl sulphate method to avoid hazardous cyanide waste. The immature cell information channel already used in the SE-9000 is also installed in the XE-2100. Leukocyte differential, enumeration of NRBCs and reticulocyte analysis are performed using new reagents, and cellular characteristics are detected by semi-conductor laser. Details of the new principle of determination used by the XE-2100 are not yet available due to an ongoing patent application, but the limited information we were able to obtain is as follows.

Leukocytes are suspended in a lysing reagent in the instrument, the leukocyte membrane is slightly damaged, and then the cells are stained with the new reagent '4DIFF', which contains a dye that binds to DNA and RNA. On a scattergram, neutrophils, lymphocytes, monocytes and eosinophils are visualized as distinctive clusters on the axes of fluorescence intensity and internal structures of cells treated with the reagent (figure 1, left top). Basophils are detected separately from other leukocytes (figure 1, right top) using a pH3 reagent, e.g. SF-3000 [1]. In order to detect NRBC, the cells are first lysed with a pH3 reagent with a low osmotic pressure of 50 mOsmol/l, and then stained with 'NRBC' reagent containing another DNA-binding dye to detect NRBCs. Erythrocyte ghosts have no fluorescence and are smaller than NRBCs, and leukocytes have higher fluorescence than NRBCs. On scattergrams of fluorescence and size, NRBCs are distributed in the central area, between erythrocyte ghosts and leukocytes (figure 1, right middle). The number of NRBCs per 100 leuykocytes is shown as 'NRBC%' on reports. Reticulocyte analysis is performed using the new reagent 'RETII', which contains a dye that binds RNA and DNA. Reticulocytes and mature erythrocytes can be differentiated by fluorescence intensity (figure 1, right bottom). Platelets are distributed on the bottom left of this scattergram due to their small size and low fluorescence, and the count obtained here is used as an optical platelet count (PLT-O). The reticulocyte distribution area is divided into three regions,

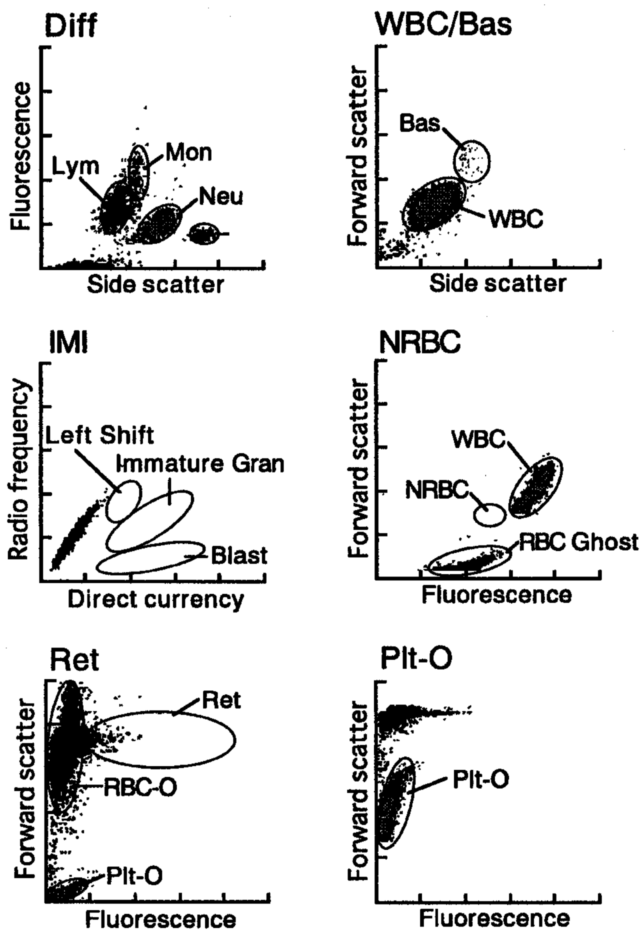

*Figure 1. Scattergrams obtained with the XE-2100.*

depending on fluorescence intensity, and the immature reticulocyte fraction can be calculated and is shown on a report. As a result, six scattergrams and two size distribution curves are obtained.

The XE-2100 was calibrated before this evaluation and checked with a quality control material daily prior to experiments.

### *Reproducibility*

Samples from healthy adults ($n = 5$) were assayed five times, and the coefficients of variation (CV) for each parameter were calculated. Normal samples had no NRBC, so the same number of samples with NRBC% more than 0% were selected for evaluation of reproducibility of NRBC determinations.

### *Carryover test*

Triplicate measurements of a high-count (a1–a3) sample followed by a low-count (b1–b3) sample were performed, and carryover effect was determined [2].

### *Stability during storage*

Samples from healthy adults ($n = 3$) were stored for up to 48 h, and the changes in obtained parameters during storage were determined. Each sample was stored in two tubes, one at 4 °C and one at room temperature (25 °C), and measured at 0, 0.5, 4, 8, 24 and 48h after blood collection. The samples stored at 4 °C were left at room temperature for 10 min before measurements. The percentage changes in parameters were calculated for each sample, with the value at 0 h set at 100%. Mean values of results for three samples were plotted.

### *Comparability to other methods*

The samples were processed on the XE-2100 and the laboratory's routine haematology analyser (SE-9000, Sysmex). The findings obtained with the two instruments were compared. For leukocyte differentials, manual differential counts were also performed on May–Grünwald–Giemsa-stained peripheral blood films prepared by the wedge methods. Medical technologists with more than 10 years of experience performed 100 cell differential counts. For comparison of differential counts, samples were selected to ensure similar percentages of morphologically normal and abnormal samples. For reticulocyte analysis, the findings obtained with the automated haematology analyzer R-3000 (Sysmex) were used for comparison.

### *Bone marrow analysis*

Bone marrow aspirates ($n = 15$) were obtained from the posterior iliac crest. The volume of aspirates ranged from 0.5 to 1.0 ml, and each sample was anticoagulated with ethylenediamine tetraacetic acid-2K. For manual counting, a 1:50 dilution of the marrow with Türk's diluting fluid was performed in a Melangeur blood-diluting pipette for white blood cells. Nucleated cells of these diluted samples were manually determined using a Buker Türk haemocytometer. Smears were prepared on glass slides, and after May–Grünwald–Giemsa staining, cells were observed under a microscope (500 count). For assay with the XE-2100, bone marrow aspirates were diluted with physiological saline (five or 10 times), due to the limited volume of aspirates obtained, and immediately assayed.

Comparisons with manual differentials were conducted for total nucleated cell count, myeloid cells and erythroid cells. White blood cell counts obtained with the XE-2100 were corrected for dilution ratios. Myeloid cells detected by the XE-2100 included neutrophils, eosinophils and basophils, and the erythroid cells detected by the XE-2100 were NRBCs. Subpopulation percentages obtained with the XE-2100 were recalculated, by addtion of NRBC count to denominators.

### Results

### *Reproducibility*

The CVs for complete blood counts varied from 0.5 to 2.9% (table 1). On comparison of the impedance and optical methods for red blood cell count and platelet count determinations, the CVs obtained by optical methods were slightly higher than those obtained by the impedance method. For leukocyte differential percentages, the CVs ranged from 1.2% for neutrophils to

*Table 1. Reproducibility test.*

| | *Mean* *(range)* | *CV* *(range)* |
|---|---|---|
| WBC | 52.1 ± 4.5 (×$10^2$/μl)<br>(47.4–59.2) | 1.3 ± 0.5 (%)<br>(0.6–1.9) |
| RBC | 415.3 ± 28.5 (×$10^4$/μl)<br>(386.4–455.0) | 0.5 ± 0.2 (%)<br>(0.2–0.6) |
| RBC-o | 419.6 ± 26.1 (×$10^4$/μl)<br>(397.6–460.2) | 1.1 ± 0.4 (%)<br>(0.5–1.5) |
| Hgb | 14.2 ± 1.6 (g/dl)<br>(13.0–17.0) | 0.5 ± 0.1 (%)<br>(0.4–0.7) |
| Hct | 36.6 ± 3.5 (%)<br>(34.0–42.7) | 0.9 ± 0.2 (%)<br>(0.7–1.2) |
| MCV | 88.1 ± 6.1 (fl)<br>(79.3–93.9) | 0.8 ± 0.2 (%)<br>(0.6–1.0) |
| MCH | 34.0 ± 2.4 (pg)<br>(31.6–37.3) | 0.7 ± 0.2 (%)<br>(0.5–0.9) |
| MCHC | 38.6 ± 1.2 (g/dl)<br>(37.4–39.9) | 1.1 ± 0.2 (%)<br>(0.7–1.3) |
| Plt | 24.7 ± 8.1 (×$10^4$/μl)<br>(17.9–38.1) | 1.9 ± 0.8 (%)<br>(1.1–3.3) |
| Plt-o | 20.7 ± 8.8 (×$10^4$/μl)<br>(13.6–35.4) | 2.2 ± 1.0 (%)<br>(0.9–3.3) |
| RDW-SD | 44.5 ± 7.2 (fl)<br>(37.5–56.5) | 0.8 ± 0.3 (%)<br>(0.3–1.1) |
| RDW-CV | 13.7 ± 1.5 (%)<br>(12.8–16.4) | 0.5 ± 0.1 (%)<br>(0.4–0.7) |
| PDW | 11.6 ± 1.0 (fl)<br>(9.9–12.6) | 2.3 ± 1.0 (%)<br>(0.8–3.2) |
| MPV | 10.2 ± 0.6 (fl)<br>(9.1–10.7) | 0.9 ± 0.5 (%)<br>(0.4–1.7) |
| P-LCR | 26.8 ± 5.1 (%)<br>(18.2–31.4) | 2.9 ± 1.0 (%)<br>(1.9–4.1) |
| PCT | 0.2 ± 0.0 (%)<br>(0.2–0.3) | 2.3 ± 0.4 (%)<br>(1.8–3.0) |
| Neu% | 63.8 ± 4.1 (%)<br>(58.9–69.1) | 1.2 ± 0.6 (%)<br>(0.3–1.7) |
| Lym% | 22.5 ± 6.7 (%)<br>(13.9–29.6) | 4.3 ± 1.4 (%)<br>(2.3–5.9) |
| Mon% | 10.6 ± 3.6 (%)<br>(5.5–15.7) | 6.0 ± 2.3 (%)<br>(3.3–9.7) |
| Eos% | 2.1 ± 1.5 (%)<br>(0.6–4.7) | 17.0 ± 17.8 (%)<br>(3.2–47.7) |
| Bas% | 1.0 ± 0.2 (%)<br>(0.7–1.3) | 18.3 ± 12.7 (%)<br>(4.3–34.9) |
| NRBC% | 2.1 ± 0.8 (%)<br>(1.3–3.3) | 9.4 ± 3.3 (%)<br>(6.0–13.2) |

18.3% for basophils. Samples used to evaluate NRBC reproducibility had a mean NRBC% of 2.1% and a mean CV of 9.4%.

### *Carryover*

The mean values of high and low white blood cell counts were 234.6 and 3.3 (×$10^3$/μl); those for red blood cell count were 614.0 and 203.7 (×$10^4$/μl); and those for platelet count were 107.7 and 3.8 (×$10^4$/μl).

Carryover for white blood cell count was 0%, while that for red blood cell count was 0.24% and that for platelet count was 0.67%.

### *Stability during storage at 4 °C and room temperature*

Complete blood count parameters were stable for up to 8 h of storage regardless of temperature (figure 2). After 24 h of storage, platelet count tended to decrease, especially at room temperature. Mean corpuscular volume was increased after 24 h of storage at room temperature, but not at 4 °C. The percentage changes up to 48 h were within 10% for %neutrophils, %lymphocytes and %monocytes at room temperature. The changes in %basophils appeared to be high, but the actual changes in percentages in differentials of basophils were at most 0.9% for 48 h of storage at room temperatue. These changes were smaller at 4 °C, and even at 48 h after blood collection, the percentage changes for %neutrophils and %lymphocytes were within 3%.

### *Comparability to other methods*

The correlations for complete blood count parameters ($n = 210$) between the XE-2100 and SE-9000 analysers are shown in table 2. Correlation coefficients ranged from 0.867 to 0.999. Leukocyte differential findings obtained with the XE-2100, SE-9000 and manual method were compared for 126 samples. The correlation coefficients for %neutrophils, %lymphocytes, %monocyte and %eosinophils were over 0.8 among these three methods (figures 3–5). For %basophils, the $r$ values among the three methods were above 0.5, although the range of variation for %basophils was narrow. Reticulocyte percentages obtained with the XE-2100 also correlated well with those obtained with the R-3000 (figure 6). Samples with more than 0 count NRBCs per 100 white blood cells by either manual or XE-2100 methods were used for comparison ($n = 77$). The NRBC% obtained with the XE-2100 correlated well with the manual count, with an $r$ of 0.859.

### *Quantitative analysis of bone marrow aspirates*

The total nucleated cell count determined manually and the corrected white blood cell count obtained with the XE-2100 correlated well (figure 7). High correlations were found between manual counting and XE-2100 determination for percentages of myeloid and erythroid cells.

### Discussion

The present evaluation showed that the accuracy of determination of complete blood counts, leukocyte differentials and reticulocyte percentages by the XE-2100 were satisfactory. Although automated leukocyte differential counting is now possible, it has remained difficult to differentiate NRBCs from leukocytes, because NRBCs are often distributed near lymphocytes on scattergrams and discrimination of those two types of cells is not easy. With the XE-2100, as a consequence of treatment with lysing reagent and staining dye,

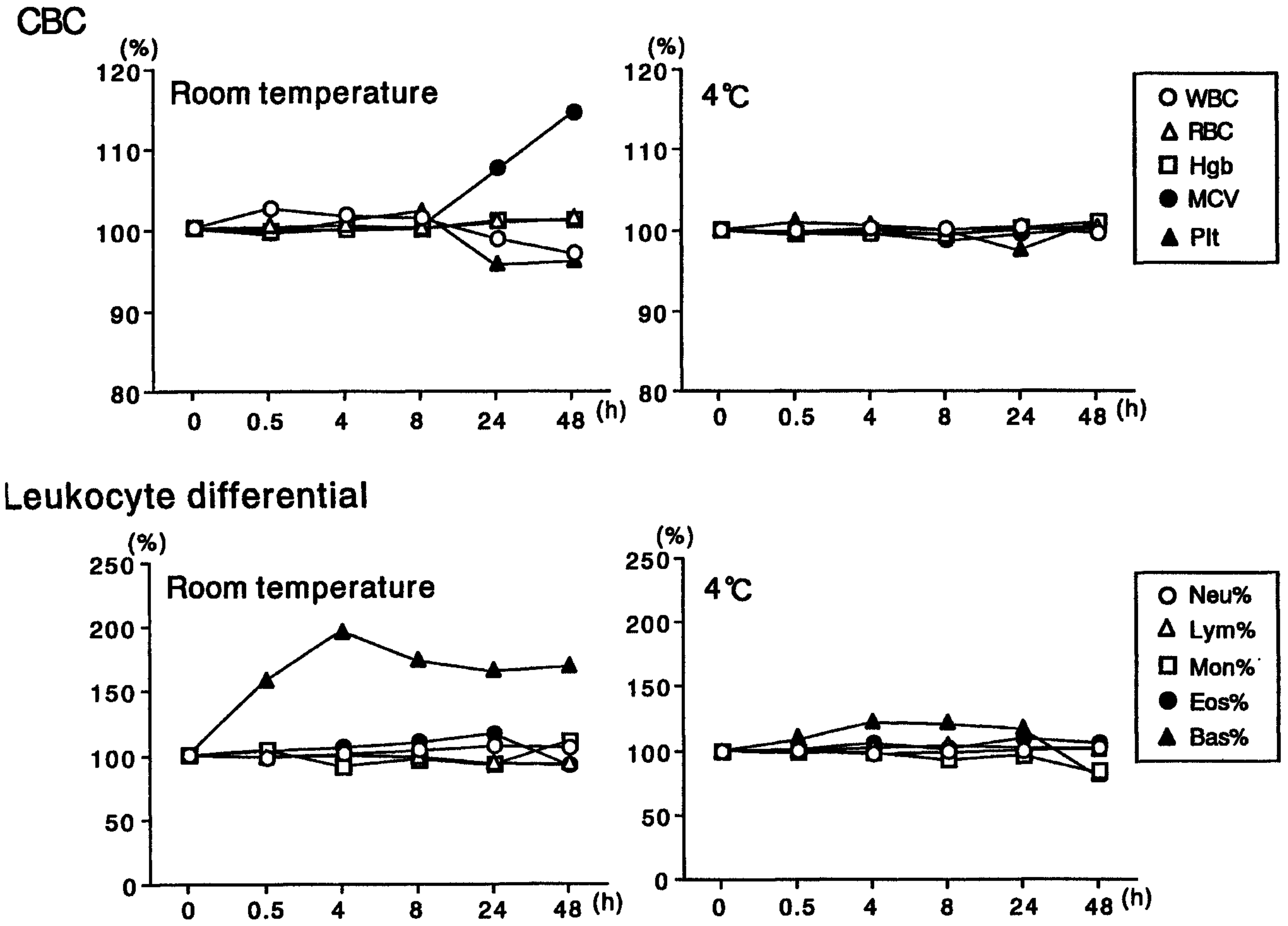

*Figure 2. Stability test.*

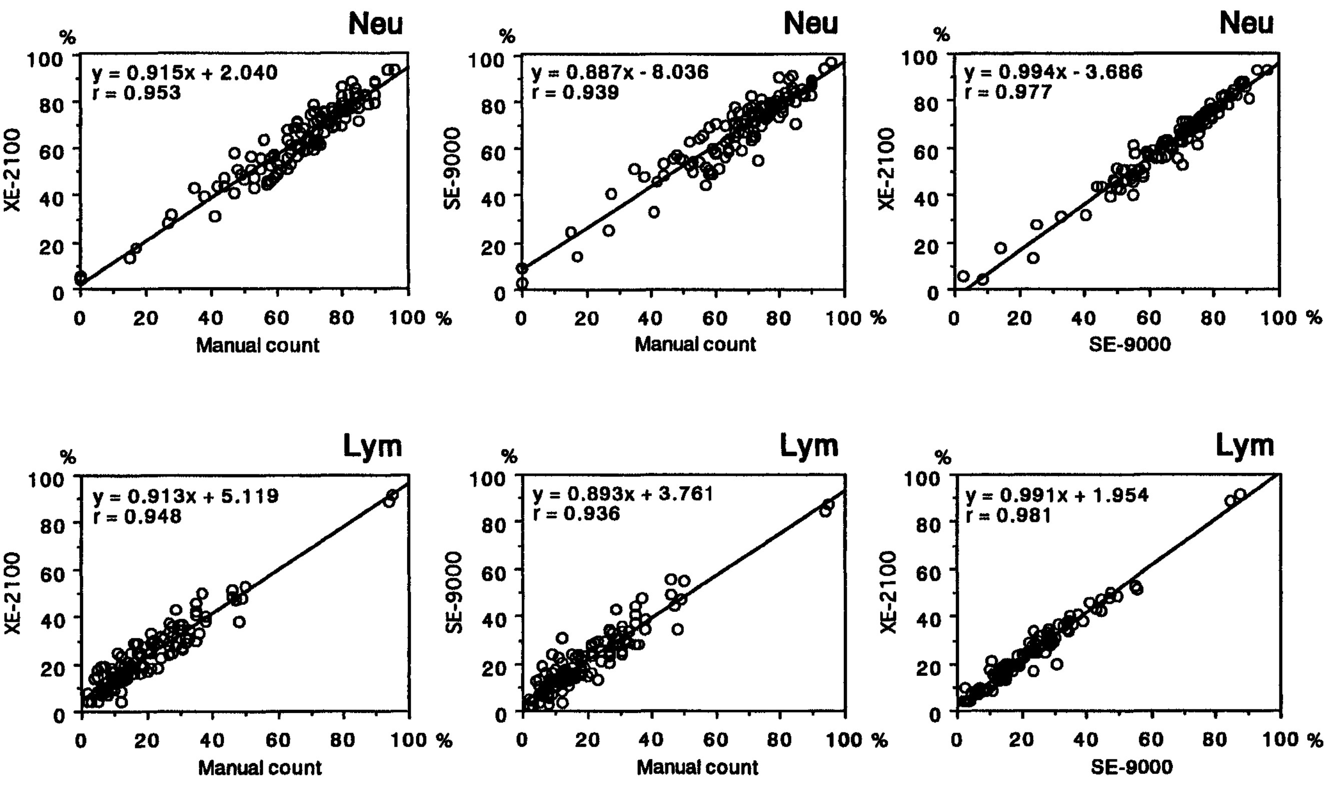

*Figure 3. Correlations for %neutrophils and %lymphocytes among manual method, XE-2100 and SE-9000.*

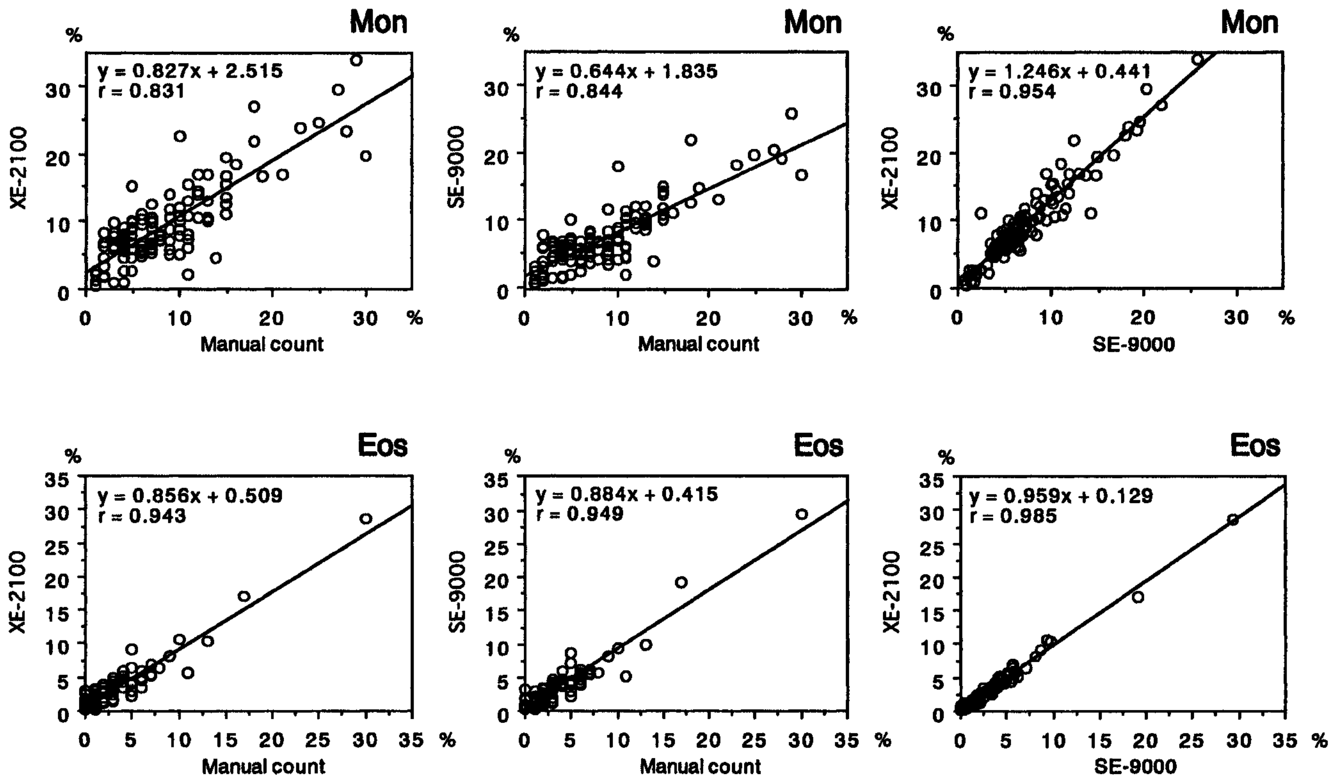

*Figure 4. Correlations for %monocytes and %eosinophils among manual method, XE-2100 and SE-9000.*

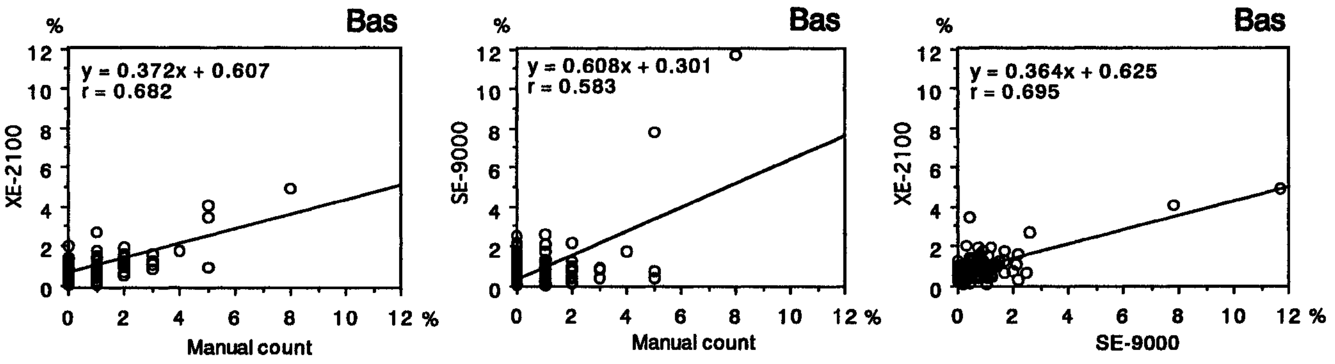

*Figure 5. Correlations for %basophils among manual method, XE-2100 and SE-9000.*

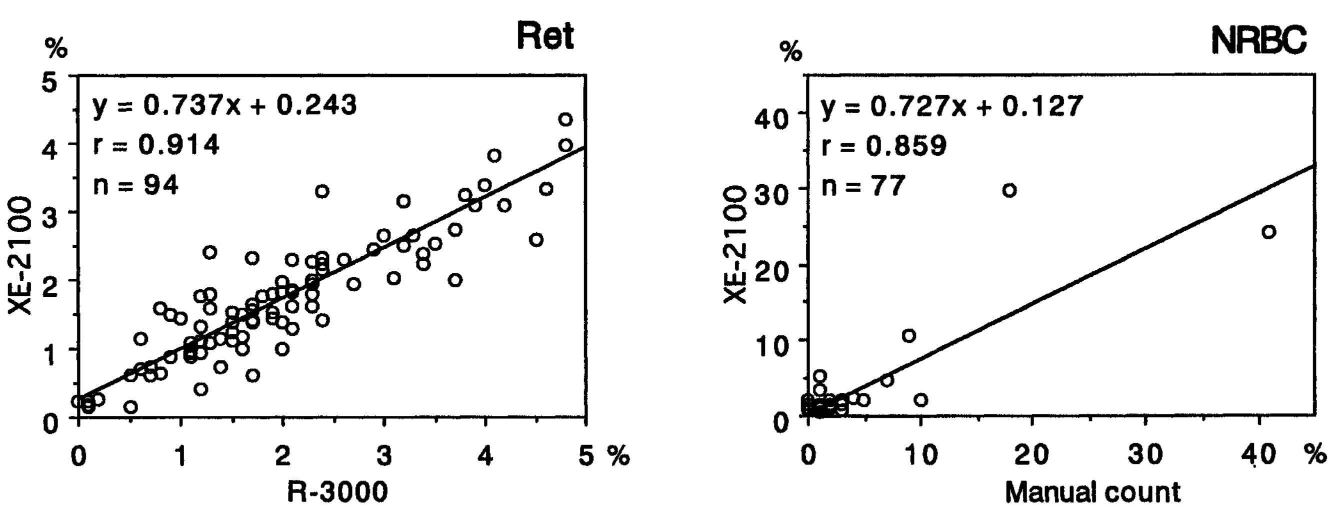

*Figure 6. Correlations for reticulocyte percentage and nucleated red blood cell count.*

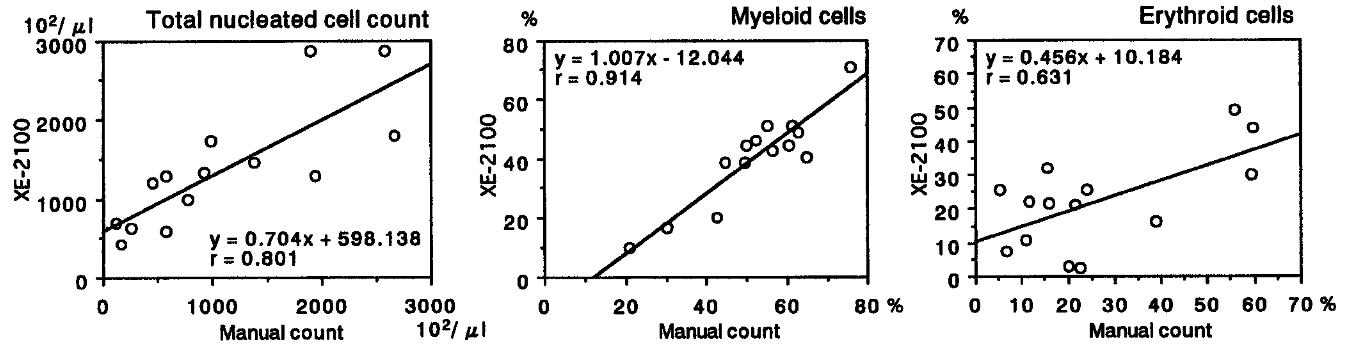

*Figure 7. Correlations for total nucleated cell count, percentages of myeloid cells and percentages of erythroid cells in bone marrow aspirates.*

*Table 2. Correlations between XE-2100 and SE-9000 for complete blood counts.*

| | | $X = SE$-9000 | $Y = XE$-2100 | *Equation* $y = ax + b$ | $r$ |
|---|---|---|---|---|---|
| WBC ($10^2/\mu l$) | mean ± SD | 91.5 ± 139.7 | 87.2 ± 141.3 | $y = 1.010x - 5.307$ | 0.999 |
| | range | (10.2–946.0) | (10.0–973.9) | | |
| RBC ($10^4/\mu l$) | mean ± SD | 345.2 ± 74.3 | 307.0 ± 64.0 | $y = 0.858x + 10.803$ | 0.996 |
| | range | (6.1–15.7) | (6.2–15.5) | | |
| Hgb (g/dl) | mean ± SD | 10.4 ± 2.0 | 10.3 ± 2.0 | $y = 0.963x + 0.209$ | 0.995 |
| | range | (171.0–552.0) | (6.2–15.5) | | |
| Hct (%) | mean ± SD | 31.9 ± 6.1 | 27.6 ± 5.2 | $y = 0.851x + 0.393$ | 0.993 |
| | range | (18.0–47.0) | (15.2–41.3) | | |
| MCV (fl) | mean ± SD | 93.3 ± 7.9 | 90.4 ± 7.5 | $y = 0.936x + 3.072$ | 0.979 |
| | range | (64.8–121.1) | (62.3–113.8) | | |
| MCH (pg) | mean ± SD | 30.5 ± 3.2 | 33.7 ± 3.5 | $y = 1.093x + 0.318$ | 0.990 |
| | range | (17.7–41.9) | (19.7–45.3) | | |
| MCHC (g/dl) | mean ± SD | 32.7 ± 1.3 | 37.2 ± 1.7 | $y = 1.096x + 1.402$ | 0.867 |
| | range | (27.3–36.1) | (31.6–42.0) | | |
| Plt ($10^4/\mu l$) | mean ± SD | 23.5 ± 16.2 | 22.9 ± 15.2 | $y = 0.932x - 1.012$ | 0.995 |
| | range | (1.4–94.3) | (1.5–84.7) | | |

$n = 205$.

leukocytes, NRBCs and erythrocytes have different sizes and fluorescence intensities, and cell clusters distribute to different areas on scattergrams and are therefore easy to discriminate. Quantitative data on NRBCs can thus be obtained. The precision of NRBC determination with the XE-2100 was excellent, and similar to that for the same percentages of other leukocyte subpopulations. It is advantageous for routine laboratories to be able to obtain such precise measurement for NRBCs, as slight changes in NRBC counts in patients can therefore be followed. The accuracy of the XE-2100 was satisfactory at NRBC counts of less than 10% obtained by the manual 100 eye count method ($n = 74$); no case was out of the range of Rümke's 95% confidence limit [3]. Only the three cases with the highest NRBC count were outside the 95% confidence range, and all three were cases of leukaemia (L2 and two cases of myelodysplastic syndrome), in which the presence of abnormal cells may have been the cause of the discrepancy between XE-2100 and manual counts. The NRBC scattergram clearly revealed the presence of NRBCs, as illustrated in figure 8. This was a case of sickle cell anaemia, and the scattergram also shows an abnormally long area of distribution of erythrocyte ghosts. Laharrague *et al.* [4] reported that

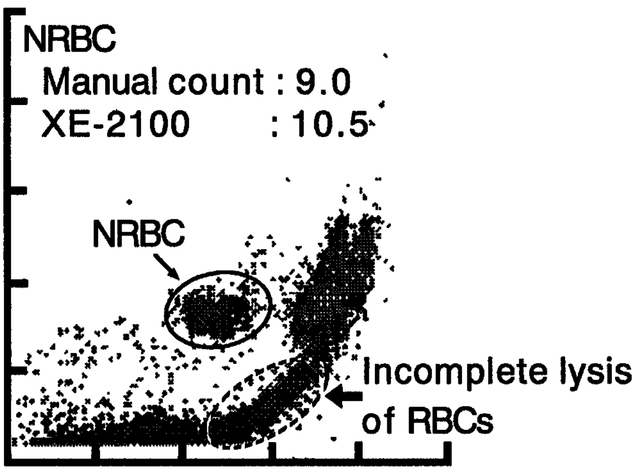

*Figure 8. NRBC scattergram for a case of sickle cell anaemia.*

peculiar patterns of areas of eryhtrocyte ghosts were frequently found for haemoglobinopathies and cases of severe hepatic dysfunction using the Sysmex NE-8000, and noted that the abnormal scattergrams obtained were due to incomplete lysis of erythrocytes. The unusual scattergram in figure 8 may also have resulted in part from the incomplete lysis of erythrocytes, and the NRBC scattergram should be useful in revealing such abnormalities. It should also be noted that enumeration of NRBC does not provide just one additional parameter for specimens—it also makes possible more accurate leukocyte counting uninfluenced by the presence of NRBCs, which may or may not have been included in leukocyte counts by previous instruments.

Another recently developed haematology analyser, the Cell Dyn 4000, which can also count NRBCs, has been used for automated bone marrow analysis [5, 6]. The degree of comparability with manual differentials of the two instruments did not differ much, and both instruments tended to underestimate erythroid cell counts. For both instruments, the reagents used for assay and the algorithm for differential counts are now designed for analysis of peripheral blood, and there is room for improvement of bone marrow analysis. However, it is advantageous to laboratories to rapidly obtain numerical data on bone marrow aspirates by precise and objective methods. Because the CVs of subpopulation percentages in bone marrow aspirates (data not shown) were similar to those for peripheral blood, precise analysis was assured for automated bone marrow analysis.

Although platelet counts can now be obtained with sufficient reliability with conventional automated haematology analysers, automated methods are not without limitations. Results for a few abnormal cases must be confirmed by methods not based on the impedance principle, as the automated method cannot differentiate platelets from small erythrocytes, erythrocyte fragments or immune complexes [7]. With the impedance method, only the size of cells can be detected, but with the optical method, more accurate determination has become possible, with discrimination of platelets from other small particles with similar sizes. Interference with large platelets and platelet aggregation is also reduced. In our evaluation, the optical method yielded platelet counts as precisely as the conventional impedance method. We did not encounter cases for which significantly inconsistent platelet counts were obtained by the two methods. With correct detection of platelets, routine laboratories can reduce the time needed to perform the tedious and time-consuming manual observations that are now sometimes required.

What can be emphasized as an advantage of XE-2100 is the possibility of stable measurement over an extended period of time. Leukocytes degenerate during storage and thresholds on scattergrams correspondingly became vague, but the XE-2100 could nevertheless differentiate each subpopulation of cells. In order to obtain correct differential counts with automated haematology analysers, storage for only 8 h, and at longest 24 h, is generally recommended. However, the XE-2100 provided stable results over 48 h of storage, and is in this respect superior to other haematology analysers presently available [1, 8]. The quality of measurements with it is not reduced by storage for long periods of time; this should be of importance to laboratories treating large numbers of samples. The XE-2100 can assay 150 samples/h for complete blood counts and leukocyte differentials, and 113 samples/h with the addition of reticulocyte analysis. This throughput is higher than that of any other analyser presently on the market, and will help to shorten turnaround times. The display and computer components of the XE-2100 are Windows-based, and are therefore very easy to operate even for individuals unfamiliar with the instrument. Sophisticated data-processing and internet communication network data-transmission protocols are installed in the analyser. Communication, looking at the same display, is possible among individuals working at long distances.

There are financial issues laboratories must face, and the above advantages should potentially permit savings in the form of less stat and duplicate testing, and more efficient use of clinician time. Clinically, the XE-2100 should make possible advanced detection of immature cells. The immature cell information channel, the so-called IMI channel, installed in the XE-2100 is now used to detect haematopoietic progenitor cells [9], and quantitative evaluation of immature granulocytes may be possible in the near future.

In conclusion, the XE-2100 analyser will improve clinical laboratory productivity and efficiency, and the more detailed automated examination it enables will improve both the quality and quantity of laboratory testing.

### References

1. Tsuda, I., Sagawa, H., Takubo, T., Kawai, S. and Tatsumi, N., *Journal of Automatic Chemistry*, **18**, (1996), 163.
2. Broughton, P. M., Gowenlock, A. H., McCormack, J. J. and Neill, D. W., *Annals of Clinical Biochemistry*, **11**, (1974), 207.
3. Rümke, C. L., in *Differential Leukocyte Counting*, Ed. Koepke, J. A. (Skokie, IL: College of American Pathologists, (1977), Vol. 4, 39.
4. Laharrague, P. F., Corberand, J. X., Fillola, G. and Cambus, J. P., *Hemoglobin*, **16**, (1992), 291.
5. D'Onofrio, G., Zini, G., Tommaswi, M., Laurenti, L., van Hove, L., Diga, S. and Leone, G., *Laboratory Hematology*, **4** (1998), 71.
6. Sakamoto, C., Yamane, T., Ohta, K., Hino, M., Tsuda, I. and Tatsumi, N., *Acta Haematologica* (in press).
7. Ault, K. A., *Laboratory Hematology*, **2**, (1996), 139.
8. Tatsumi, N., Tsuda, I., Shimizu, A., Watanabe, K. and Matuno, K., *Laboratory Hematology*, **2**, (1996), 157.
9. Takekawa, K., Yamane, T., Suzuki, T., Hino, M. and Tatsumi, N., *Acta Haematologica*, **98**, (1997), 54.

